# A Compact Regulatory Logic Governs Fibroblast Cell State Plasticity in Fibrosis

**DOI:** 10.64898/2026.10.09.757677

**Published:** 2026-10-16

**Authors:** Ada M. Zhu, Yeting Li, Haiwei Zhang, Brindha Rathinasabapathi, Bastien Cimarosti, Rangarajan Nadadur, Vee Xu, Yu Huang, Yuqian An, Kirsten Auclair, Alicja Mahr, Maya U. Sheth, Clara Youngna Lee, Chen Wang, Tracy Yamawaki, Angelo Pelonero, Arun Padmanabhan, Sanjeev S. Ranade, Jesse M. Engreitz, Brandon Ason, Chi-Ming Li, Simon Jackson, Junedh M. Amrute, Pawel F. Przytycki, Michael Alexanian

## Abstract

Fibroblast activation drives organ failure across human diseases, yet its regulatory basis remains largely undefined. Here, we repressed 280 conserved disease-activated enhancers using CRISPR droplet sequencing (CROP-seq) in ∼550,000 fibroblasts, constructing a regulome of 15 gene programs governing the profibrotic state. Systematic TF perturbation converged on MEOX1 as a central regulator, and fibroblast-specific *Meox1* deletion in mice improved cardiac function, reduced fibrosis, and rewired profibrotic fibroblasts toward quiescence in heart failure. Effector gene perturbation in human cardiac fibroblasts revealed distinct regulators of contractility, migration, and inflammatory signaling, and these programs were conserved across profibrotic states in human lung, liver, and skin. Simultaneous repression of just six enhancers using a multiplexed dCas12a-KRAB system collapsed the profibrotic network, suppressed migration and contraction, and rewired fibroblast paracrine signaling to attenuate macrophage inflammation. These findings define the regulatory logic of fibroblast plasticity and establish a new framework for reprogramming profibrotic cell states in disease.

